# Training resources on open and responsible research and innovation (Open RRI): gaps, quality and opportunities to advance training

**DOI:** 10.12688/openreseurope.23510.1

**Published:** 2026-06-23

**Authors:** Cristina Lagido, Kristian Nielsen, Gitte Kragh, Pascal Flohr, Louise Bezuidenhout, Loek Brinkman, Agnese Baini, Chiara Saviane, Asya Salnikova, Adam Brandstetter-Kunc, Giacomo Consolini, Antónia Correia, Paula Moura, Maya Fedeli, Jonathan England, Shanmugasundaram Venkataraman, Aslak Skarvøy, Teodora Konach, Borana Taraj, Katharina Koller, Ilse Marschalek, Maria Schrammel, Sanja Jurkovic, Bojan Macan, Alen Vodopijevec, Brian Cahill, Andrea Giraldo Sevilla, Nico Pitrelli, Alessio Livio Spera, Claudia Iasillo, Pedro Príncipe, Noemi De Lorenzo, Muki Haklay

**Affiliations:** 1 Aarhus University, Aarhus, Denmark; 2 Data Archiving and Networked Services, The Hague, The Netherlands; 3 Leiden University, Leiden, The Netherlands; 4 SISSA - Scuola Internazionale Superiore di Studi Avanzati, Trieste, Italy; 5 European Science Foundation, Strasbourg, France; 6 Universita Vita-Salute San Raffaele, Milan, Italy; 7 Universidade do Minho, Braga, Portugal; 8 OpenAIRE, Athens, Greece; 9 EARMA - European Association of Research Managers and Administrators, Brussels, Belgium; 10 ZSI - Zentrum für Soziale Innovation, Vienna, Austria; 11 Ruđer Bošković Institute, Zagreb, Croatia; 12 SciLink Foundation, Amsterdam, The Netherlands; 13 LPI - Learning Planet Institute, Paris, France; 14 APRE - Agenzia della Promozione della Ricerca Europea, Rome, Italy; 15 University College London, London, England, UK

**Keywords:** Open and Responsible Research and Innovation (Open RRI); Training resources; Open RRI education; Quality assessment; Evaluation Framework; Capacity building

## Abstract

**Background:**

Open Science (OS) and Responsible Research and Innovation (RRI) are increasingly converging under the concept of Open RRI, which integrates openness with inclusivity, responsibility, and societal engagement. Strengthening researcher skills in this area requires a clear understanding of existing training provision. This study presents findings from the Horizon Europe project PATTERN, which mapped and assessed Open RRI training resources across Europe.

**Methods:**

A mixed-methods approach was applied, combining desk research, surveys, semi-structured interviews, data workshops, and mutual learning events. In total, 571 training resources were identified and analysed across eight skill areas: Open Access; FAIR Data; Citizen Science; Research Integrity; Gender, Non-discrimination, and Inclusion; Dissemination and Exploitation; Science Communication; and Management and Leadership. A subset of resources was further evaluated using defined quality criteria.

**Results:**

The mapping reveals a fragmented and uneven training landscape, with a strong predominance of introductory-level resources and limited provision for intermediate and advanced learners. Training is often generic and lacks contextualisation, with gaps in inclusivity, multilingual provision, and local adaptation. Quality assessment highlights strengths such as modular design, open licensing, and the use of active learning approaches. However, recurring challenges include limited accessibility, unclear pathways for reuse, and difficulties in implementation and long-term sustainability. Key thematic gaps include intersectionality, policy engagement, supervision ethics, and the integration of indigenous and local knowledge.

**Conclusions:**

Despite growing availability of training resources, significant gaps remain in scope, depth, and inclusivity. Addressing these challenges requires improved coordination, expansion of advanced and context-sensitive training, enhanced multilingual accessibility, and stronger integration into curricula and institutional practices to support sustainable Open RRI capacity building.

## Plain Language summary

This study examines the training available to help researchers work in more open, ethical, and socially responsible ways—a combined approach known as Open and Responsible Research and Innovation (Open RRI). We analysed 571 training resources from across Europe, covering topics such as open access, FAIR data, citizen science, research integrity, gender and inclusion, science communication, and management and leadership.

To understand the current landscape, we used a mix of methods including desk research, interviews, surveys, data workshops, and mutual learning events. This allowed us to identify what training exists, assess its quality, and highlight where important gaps remain. We found that most training is introductory, often limited in depth, and primarily available in English. Few resources support advanced learning, leadership development, or skills related to inclusivity, policy engagement, or interdisciplinary collaboration. Training also varies widely in accessibility, licensing, and ease of reuse.

Despite these challenges, we identified many high-quality, openly licensed materials that can be adapted and built upon. To strengthen the field, we recommend improving coordination between training providers, offering resources in multiple languages, ensuring long-term sustainability, and embedding Open RRI training more firmly within institutional development programmes.

## Introduction

Responsible Research and Innovation (RRI) and Open Science (OS) are two coexisting ambitions in the practice of research and innovation in the present day. RRI aims to align the research and innovation processes and outcomes with societal values, needs and expectations ( Schuijff & Dijkstra, 2020). This demands anticipation, responsibility, and responsiveness from all involved in the research process. It also acknowledges the broad stakeholder basis of research and innovation, including researchers, citizens, policy makers, business, third sector organisations, and other societal actors. RRI gained traction in Europe through the Horizon 2020 Framework Programme (H2020) under the Science with and for Society (SwafS) initiative ( Owen et al., 2012; Tabarés et al., 2022). The European Commission emphasises five dimensions of RRI to translate the concept into practice—open access, ethics, science education, public engagement, and gender equality—supported by integrated governance ( Schuijff & Dijkstra, 2020).

In turn, OS aims to make multilingual scientific knowledge openly available, accessible and reusable, fostering collaboration and information sharing for societal and scientific benefit ( OECD, 2015; UNESCO, 2021). It also seeks to promote citizen science by involving societal actors beyond the traditional scientific community in the creation, evaluation, and dissemination of scientific knowledge. While both approaches of RRI and OS share a commitment to inclusivity and sustainability, OS has traditionally been more focused on technical optimisation, such as open access, open data, and the use of digital tools, whereas normative concerns and democratic deficits tend to be a main concern of RRI ( Shelley-Egan et al *., *2020). Embracing transformative change in research and innovation culture, the European Commission has increasingly supported efforts to integrate the RRI and Open Science approaches. This ambition is taken up in the Horizon Europe-funded project Piloting open and responsible Activities and Trainings Towards the Enhancement of Researchers Networks (PATTERN), which adopts the evolving concept of Open RRI ( CORDIS, 2025).

Operationalising Open RRI presents challenges such as the limited knowledge of the concept but also misplaced career concerns ( Marschalek et al., 2017; McKiernan et al., 2016). To address this, it is essential to provide researchers at all career stages with training in Open RRI awareness and skills ( El Idrissi et al., 2023; O’Carroll et al., 2017). Efforts to harmonise researcher development across the European Research Area have led to the emergence of common frameworks, such as the recently published European Competence Framework for Researchers, known as ResearchComp ( European Commission, 2023). This framework outlines competencies and skills for researchers across four levels—foundational, intermediate, advanced, and expert—offering a set of expectations that can be used for a wide range of goals. It can help define minimum training requirements for early-career researchers, support accreditation and evaluation of researcher training programmes, and act as a guidance for the development of job descriptions. From the perspective of this paper, the identification of high-quality training can facilitate the implementation of frameworks such as ResearchComp and assist universities and other research performing institutions in preparing their researchers for future career challenges and promoting education for sustainability ( CoARA, 2022).

In Europe, much of the training in Open RRI has been actively supported not only through EU-funded projects but also by research infrastructures such as the European Open Science Cloud (EOSC). The EOSC provides a federated digital environment for open access to datasets, tools, and services ( Commission High Level Expert Group on the European Open Science Cloud, 2016). The EOSC Synergy project (2019–2022) was important in promoting best practices in FAIR (Findable, Accessible, Interoperable, Reusable) data and open science within EOSC, while the establishment of EOSC-related curricula and training have been the focus of Skills4EOSC (2022–2025). Earlier efforts of note include the projects Facilitate Open Science Training for European Research (FOSTER, 2014–2016) and FOSTER+ (2017–2019), which created a collection of well-used learning materials for open science and open access now available on the e-learning platform OpenPlato ( Bezjak et al., 2018; Schmidt et al., 2016).

Further pillars of Open Science and RRI, have been supported through the dedicated SwafS programme under H2020. It focused on projects to strengthen various areas, including citizen science, gender equality, science education, research ethics and integrity, and science communication. Among these were QUality and Effectiveness in Science and Technology communication (QUEST) and Global Science Communication and Perception (GlobalSCAPE) in science communication ( Mannino et al *., *2021; Roche et al., 2024); Supporting Sustainable Institutional Changes to Promote Citizen Science in Science and Technology (TIME4CS) ( Fedeli et al., 2025; Nielsen & Kragh, 2023); Gender Equality in Academia (GE Academy); European Network of Research Ethics and Research Integrity (ENERI) ( Löfström et al., 2025); and Responsible Open Science in Europe (ROSiE) ( Lindemann & Häberlein, 2023).

This paper presents findings from the Piloting open and responsible Activities and Trainings Towards the Enhancement of Researchers Networks (PATTERN) project, which aims to enhance Open RRI practices through an integrated training, assessment, and intervention approach. Involving 19 institutions across 13 European Research Area countries, and building on SwafS results, the project consolidated existing evidence of researcher training in Open RRI by mapping, analysing gaps, and assessing the quality of training on eight key transferable skills: Open Access; FAIR Data; Citizen Science; Research Integrity; Gender, Non-discrimination, and Inclusion in Research; Dissemination and Exploitation of Results; Science Communication (for media and policymakers); and Management and Leadership. The eight skill areas were selected based on their centrality to Open RRI, and their relevance for equipping researchers with the skills needed to address complex societal challenges across diverse institutional and national contexts.

This study serves a dual purpose: (1) to identify gaps in current Open RRI training resources to inform the development of new training materials, and (2) to assess the quality of current Open RRI training resources to promote their further dissemination and reuse. To this end, we adapted a quality framework from EU-Citizen.Science, a H2020-SwafS project that created a training platform for citizen science, citizenscience.eu ( Fraisl et al., 2020). Our gap analysis and quality assessment were underpinned by a comprehensive mapping of training resources using a mixed-methods approach ( Lagido, Nielsen et al., 2024b). The study’s novelty lies in identifying training gaps and applying a citizen science-based quality framework to assess and enhance Open RRI resources. We aim to support researchers at all career stages in integrating Open RRI into daily practice.

## Materials and methods

The collection of data on training resources took place through desk research, collaborative knowledge gathering in the form of five data workshops, 12 interviews, a survey, and three mutual learning events. This mixed methods approach was appropriate for capturing both the breadth and depth of the training landscape: while desk research and the survey provided a broad overview of existing resources and perceived needs across the European Research Area, the data workshops and interviews offered context-rich insights into local practices, challenges, and quality considerations. The mutual learning events further supported triangulation by enabling critical reflection and consensus-building among diverse stakeholders.

The data workshops were focused, 2-hour online sessions involving project partners, each dedicated to one or more of the eight skill areas identified in PATTERN. During these workshops, participants collaboratively identified relevant training resources, assessed their basic characteristics (e.g., target audience, language, delivery format), and discussed challenges related to discoverability, accessibility, and alignment with Open RRI goals. This approach facilitated distributed expertise sharing while ensuring coherence in data collection.

To gain qualitative insights into the design, delivery, and evaluation of training resources, semi-structured interviews were conducted with 12 participants, including trainers, pedagogical specialists, and representatives from other EU-funded Open RRI-related projects. Each interview lasted 45–60 minutes and followed a flexible guide focused on quality standards, challenges in training implementation, and effective practices. All interviews were conducted online, recorded with consent, and subsequently transcribed and summarised. Thematic coding of the summaries contributed to identifying key trends and informed the quality assessment process.

An online survey was distributed among PATTERN partners and their extended networks to collect systematic input on existing training offers, perceived training needs, and examples of good practice. The survey received 135 complete responses and an additional 248 partially completed responses. Around two-thirds of the respondents were based in public research institutes or universities, primarily located in Europe. In addition to capturing general trends and needs across the eight skill areas, respondents also submitted references to training resources they had developed or used. These submissions resulted in the identification of 48 additional training resources, many of which were subsequently assessed as part of the quality review process.

To foster a shared understanding of quality and stimulate cross-sector dialogue, the project organised three mutual learning events (MLEs). The first event was held internally with PATTERN partners and focused on case-based reflection to develop a common quality framework. The second and third MLEs invited external stakeholders, including trainers, pedagogical experts, and members of other Open RRI-focused EU projects, to exchange perspectives and practices. Moreover, the third MLE had a reach beyond Europe. These sessions provided space to test and refine preliminary findings and assessment approaches. Additionally, a participatory workshop was hosted at the Open Science Fair 2023 in Madrid ( Lazzeri et al., 2023), further enriching the discussion with input from the broader Open Science community. The combined outcomes of these events played a key role in aligning the project’s quality criteria with community expectations and in validating the results of the mapping and assessment phases.

### Inclusion criteria and metadata

The inclusion criteria for selecting resources were designed to ensure relevance and utility for the PATTERN project’s aims: they included alignment with the thematic dimensions of Open RRI, suitability for target audiences such as researchers, graduate students, and research support staff, and the availability of an appropriate set of descriptive metadata. These criteria were chosen to facilitate comparability, support metadata-based discovery, and ensure that the identified resources could inform both training development and strategic policy recommendations.

The metadata describing the collected resources were based on the Research Data Alliance (RDA) minimal metadata recommendations for learning resources, with some modifications ( Hoebelheinrich et al., 2022). Key elements included title, version date, author(s), language, keywords, URL, licence, access rights, resource type, learning outcomes, target audience, and expertise level. Controlled vocabularies were used where relevant to enhance standardisation and machine readability. These were based on existing vocabularies, including, but not limited to, the EOSC Training Resources Profiles ( EOSC, 2023), the EU vocabularies for Target audience, Mode of learning and assessment, Learner assessment, and Licence ( Publications Office of the European Union, 2024) as well as the formats of training resources used in the Social Science and Humanities (SSH) Training Discovery Toolkit ( Illmayer, 2023).

Additional metadata fields were incorporated, using the metadata descriptors for citizen science courses developed in the H2020-Swafs project TIME4CS ( Nielsen & Kragh, 2023) and the EOSC Training Profile documentation model ( EOSC, 2023). These included the organisation/provider, content description, content themes (skills trained)/curriculum, duration, training mode, and learner assessment. Further metadata comprised additional languages, links to EU-funded projects, scientific domain of training, predominant content resource type, and whether the resource provided a formal qualification. Several of these were based on the EOSC Training Resource model, which was in turn based on earlier projects, such as FOSTER and others. In addition, project partners contributed their expertise across PATTERN thematic areas and provided content theme descriptors to categorise the topics addressed in the training, thereby facilitating the gap analysis ( PATTERN Consortium, 2026a).

### Gap analysis and quality assessment

The PATTERN project’s gap analysis and quality assessment of training resources followed a structured, multi-step process that combined descriptive mapping with evaluative criteria grounded in previous research and stakeholder input.

*Descriptive summaries by domain experts.* Project partners with domain expertise in each of the eight PATTERN skill areas (e.g., Open Access, FAIR Data, Citizen Science, etc.) were responsible for producing descriptive summaries of the training resources collected in their respective areas. These summaries drew on data gathered through data sprints, desk research, and survey submissions and included an overview of existing platforms, types of training formats, covered topics, and accessibility features. This work was guided by a shared template, which ensured consistent documentation of metadata, coverage, and initial observations of strengths and limitations across all eight areas. Within each descriptive summary, partners also identified standout training resources that merited reuse and/or adaptation in future training events.

*Identification of resources for quality assessment.* The selection was based on initial indicators such as clarity of structure, thematic relevance, accessibility, and potential for reuse in researcher training. For feasibility reasons, only digitally available, open-access resources suitable for training researchers and research support staff were included in the quality assessment. This selection also included the curated subset of high-potential materials as identified in the descriptive summaries.

*Quality assessment criteria and application.* As already mentioned, the quality assessment applied a set of evaluative criteria adapted from the EU-Citizen.Science project ( Fraisl et al., 2020), focusing on aspects such as accessibility, readability, clarity of learning goals and pedagogical methods, ease of implementation and adaptation, and the quality of any visual or audio materials. These criteria were used to assess the usability and relevance of the selected training resources for researchers and research support staff. Building on this foundation, additional criteria, where information was available, included whether the training resource had been previously evaluated (e.g. through learner feedback or documented learning outcomes), as well as its potential or demonstrated impact on scientific practice, policy, or broader society ( Fraisl et al., 2020). Resources were rated on a five-point scale ranging from 1 (very poor) to 5 (very good). To contextualise these evaluations, the resources were also analysed using a SWOT framework (Strengths, Weaknesses, Opportunities and Threats, see Puyt et al., 2023), drawing on insights from interviews and mutual learning events, to capture their strengths, weaknesses, opportunities, and threats. This dual approach enabled a more comprehensive understanding of both the quality and the strategic value of the training resources, guiding the project’s recommendations for future training development.

## Results

### Mapped training resources

**Figure 1.**
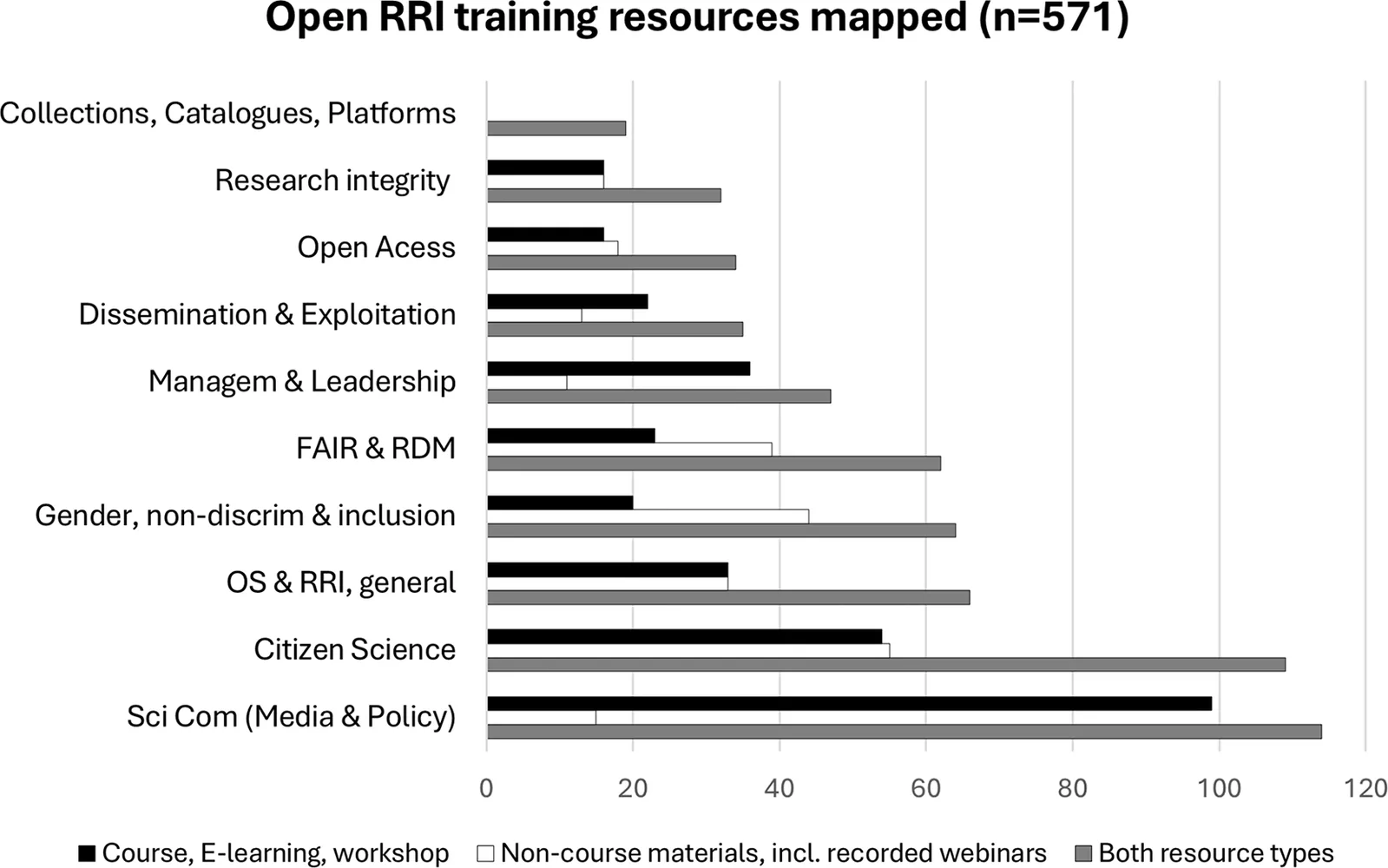
Training activities and resources mapped in the various skill areas of PATTERN. The Citizen Science and Science communication benefited from the efforts of the previous European projects EU-Citizen.Science, TIME4CS, QUEST and GlobalSCAPE. The number of training resources identified in Research Integrity are underestimated, given that the materials that are part of the Embassy of Good Science Platform were not considered individually.

PATTERN catalogued 571 training resources within the eight transferable skill target areas of Open RRI and made this information available on the Zenodo platform ( Lagido et al., 2025). Figure 1 shows the outcome of this mapping exercise in numbers. Training resources were classified into two categories: 1) courses, e-learning modules, and workshops (course-type training) and 2) non-course training materials such as recorded webinars, guidebooks, websites, etc. The distribution of these two categories was generally balanced, except in two areas. These were Gender equality, Non-discrimination and Inclusion in Research, where non-course training materials were more prevalent, and Science Communication (towards media and policymakers), where course-type training predominated.

To accommodate valuable resources that did not align neatly with the eight defined Open RRI skill areas, an additional category—Open Science and Open RRI—was introduced during the mapping process. This category captured training resources that addressed multiple or overarching aspects of Open RRI. These included materials on foundational topics such as open data, open methodologies, open reproducible research, and policy frameworks, as well as broad RRI themes like governance, ethics, and diversity. While not a core focus of the project, this category ensured that widely relevant and integrative resources were not excluded.

Citizen Science and Science Communication were among the skill areas with best coverage, reflecting extensive previous mapping efforts by the EU-Citizen.Science platform and the TIME4CS project in Citizen Science, and by QUEST and GlobalSCAPE in Science Communication. Comparatively fewer training resources appear to be available in Open Access, Dissemination and Exploitation, and Management and Leadership. Training resources in Research Integrity are numerous, contrary to what Figure 1 might suggest, as a large collection of training from the Embassy of Good Science, comprising hundreds of educational resources, was entered as a single resource.

Most identified training opportunities were in English (n = 471), followed by Italian (29), German (22), and French (17). An additional 32 resources were available in other European languages, including Croatian, Spanish, Portuguese, Finnish, Greek, Dutch, Hungarian, Swedish, Norwegian, and Slovak. This distribution likely reflects both the global dominance of English and the consortium’s mapping efforts in their respective languages.

**Figure 2.**
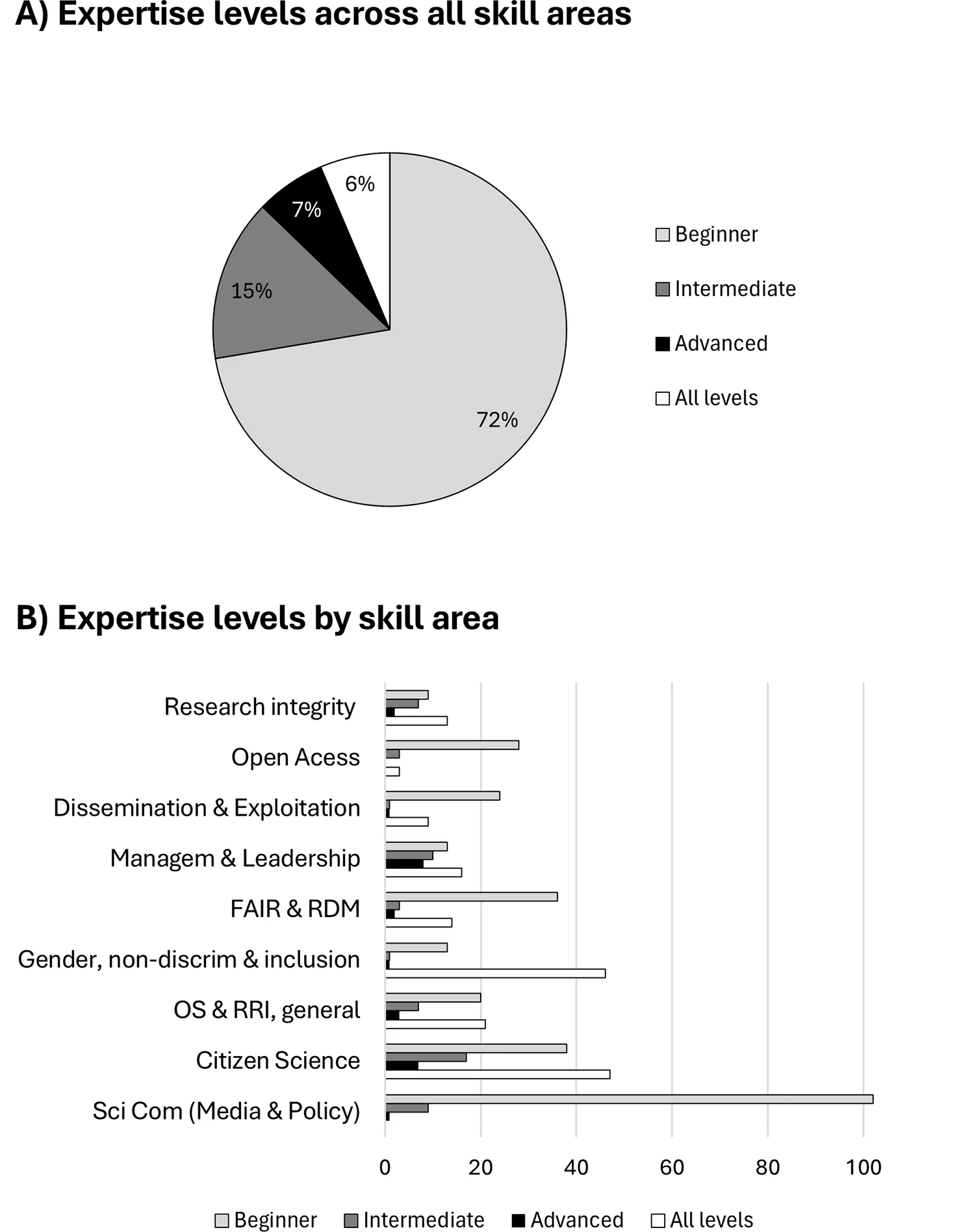
A-B: Expertise level of the mapped training in the PATTERN project: A) across all skill areas; B) per skill area within Open and Responsible Research and Innovation, including general training overarching several of these areas. The expertise levels designated were beginner, intermediate, advanced; or suitable for all.

Regarding expertise levels, most training resources targeted beginners or were designed for all skill levels ( Figure 2), highlighting a general shortage of intermediate and advanced-level offerings. However, some areas such as Citizen Science, Research Integrity, and Management and Leadership included a notable number of resources at the intermediate level, with Management and Leadership having the highest proportion of advanced training (17%). In contrast, Open Access showed a marked gap, with no resources classified at the advanced level and only three resources (9%) targeting the intermediate level.

### Quality assessment of training resources

Close to half of the catalogued resources, based on criteria of accessibility, thematic relevance, and reuse potential, underwent quality assessment. Most Management and Leadership training resources did not meet these criteria, resulting in a limited number (eight) being evaluated in this area. This is in contrast with areas like Citizen Science, where most of the training resources are openly accessible and freely available.

**Figure 3.**
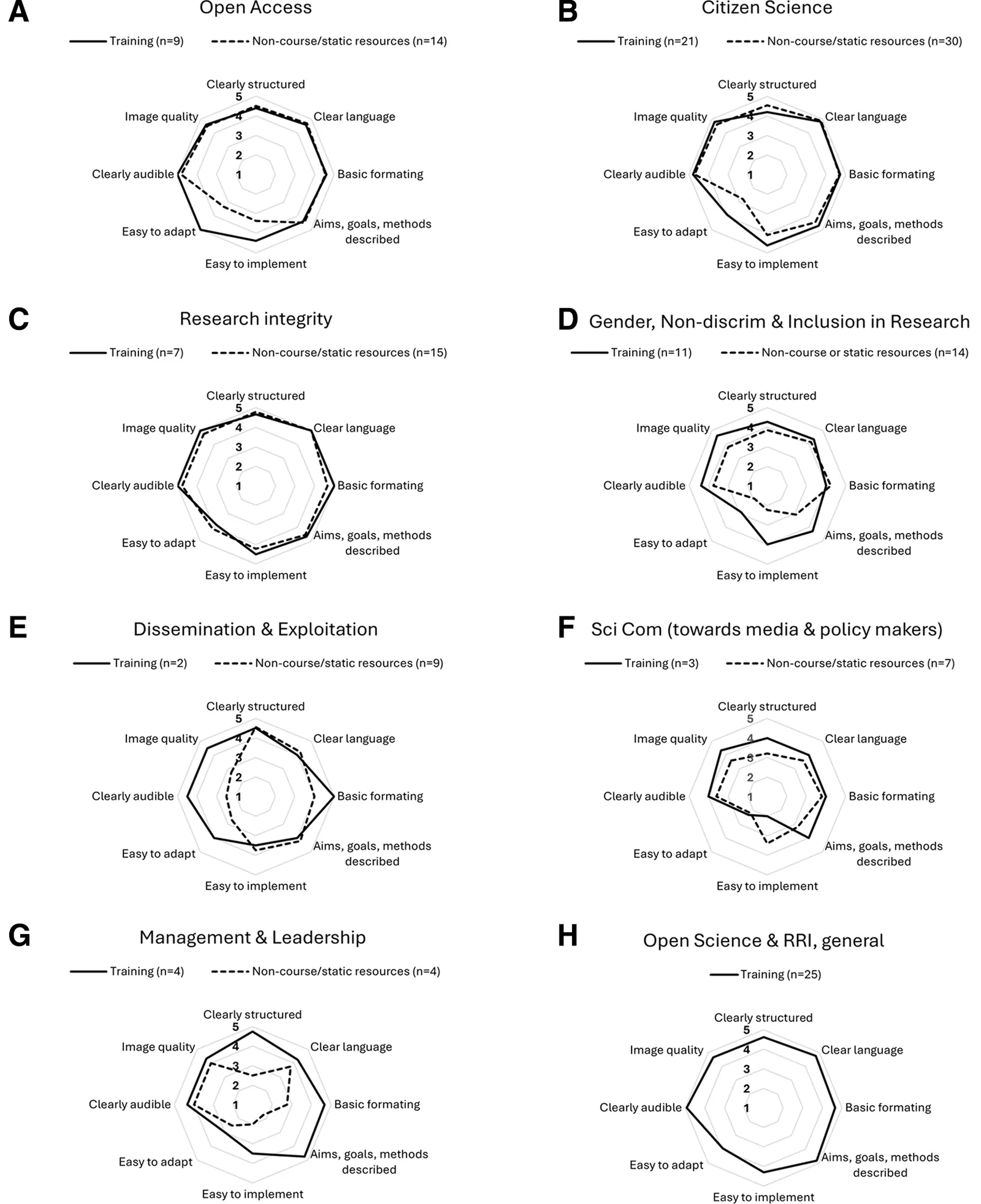
A-H: Quality assessment of digitally and freely accessible training resources in several skill areas relevant for Open RRI, according to the quality criteria of the EU-Citizen.Science project. The five-point scale was: 1, very poor, 2 poor, 3 acceptable, 4 good, and 5 very good. Training refers to courses, e-learning modules and workshops, whereas static resources refer to webinar recordings, published materials, etc.

Figure 3 presents the results of applying the EU-Citizen.Science project’s quality criteria. Overall, training resources performed well across most criteria, though scores varied by skill area. *Ease of adaptation* received the lowest evaluations, often due to unclear licensing terms, restrictive copyright, or uncertainty about reuse permissions. *Ease of implementation* was another common challenge for many resources.

Adaptation and implementation challenges vary across skill areas and often reflect broader structural and thematic issues. In Open Access training, for instance, adaptation is hindered by unclear licensing, outdated content, and limited guidance on relatively new developments like Plan S, the funder-backed scheme to require free online access to scientific literature, or diverse OA routes such as diamond publishing ( Baro & Eze, 2017; European Science Foundation, 2025). Implementation may also falter due to the lack of discipline-specific examples or regional applicability. In Gender Equality and Inclusion, resources often rely on static, non-interactive formats with limited modularity, making them harder to adapt or embed into different institutional contexts. Similarly, Science Communication training resources frequently lack clear learning outcomes and hands-on components, complicating both reuse and classroom implementation. In the area of Management and Leadership, challenges stem from generic content not tailored to research settings and an absence of practical exercises or scenario-based learning. Meanwhile, Research Integrity resources, although abundant, often face implementation issues due to limited access, lack of depth, or failure to address emerging topics such as AI ethics or interdisciplinary dilemmas. These examples highlight the need for training resources that are not only high-quality and accessible but also designed with adaptability, contextual relevance, and pedagogical flexibility in mind.

Training resources in Open Access, Open Science and Open RRI, Citizen Science and Research Integrity generally attracted good or very good evaluations across most criteria. In contrast, more static resources in Gender, Management and Leadership and Dissemination and Exploitation of Results tended to score lower in *ease of adaptation*, *ease of implementation*, and in some cases *structure*, *formatting*, *image and audio quality*, and *clarity of aims*, *goals and methods.*

Through this process, we identified key strengths and features of high-quality training resources, highlighting them for reuse ( Lagido, Kragh, et al., 2024a). High-quality training materials generally strike a balance between theoretical understanding and practical application. They provide clear, structured pathways through the content, often using step-by-step guidance, case-based learning, or real-world scenarios tailored to specific research roles or domains. These materials are typically well-organised, offering logical progression, summaries, visual cues, and explicit learning objectives to support independent learning.

Adaptability and reuse are further supported by modular design and clear licensing. Openly licensed resources with straightforward instructions make it easier for trainers and institutions to implement, translate, or tailor content for different audiences and contexts. Language that is clear, active, and free from jargon enhances accessibility and helps facilitate broader uptake, including in non-English speaking regions.

Finally, the most impactful training resources are those that are inclusive and forward-looking. They reflect attention to gender balance and representation, are available in multiple languages, and consider the needs of diverse learners. Moreover, they address emerging issues—such as AI ethics, policy engagement, or interdisciplinary collaboration—helping researchers navigate a rapidly evolving research landscape with confidence and responsibility.

### Identified gaps in Open RRI resources

The analysis of the content themes in the catalogued training—based on expert-authored summaries, quality assessments, and SWOT analyses—revealed a wide range of gaps across all eight Open RRI skill areas. These were captured as keywords (see Table 1) and clustered into six recurring thematic domains to aid interpretation:

**Table 1.** Training content gaps across the eight PATTERN transferable skill areas.

Training area	Identified content gaps
Open Access (OA)	• Limited coverage of funder requirements and policies (e.g. Plan S) • Lack of guidance on legal issues, licensing, and open peer review developments • Insufficient training on different OA publishing routes (e.g. gold, diamond) • Minimal attention to identifying and avoiding predatory journals • Underexplored discussion of OA repositories and key initiatives
FAIR Data Management	• Limited coverage in existing training materials on metadata standards, curation processes, fostering data reusability, data sensitivity and ethical data handling • Lack of training for non-specialists such as citizen scientists or researchers in non-STEM fields
Citizen Science (CS)	• Lack of integration between CS and other training areas (e.g. ethics, data, inclusion) • Minimal guidance on CS policies, fundraising strategies, and implementation roadmaps • Limited resources tailored to specific domains (e.g. humanities, health, engineering) • Scarce training on collaboration with the private sector and engaging citizens as co-creators
Research Integrity	• Limited treatment of cross-cutting issues linking RI with OS, environmental sustainability, inclusion, and AI • Insufficient focus on intellectual property, authorship, supervision ethics, power dynamics, and social consequences of misconduct • Privacy and confidentiality rarely addressed • Minimal examples beyond life and health sciences
Gender Equality, Non-discrimination, and Inclusion in Research	• Overreliance on generic content focused on GEPs, with limited attention to implementation, monitoring, and practical outcomes • Gaps in training on gender-based violence, intersectionality, and broader diversity dimensions • Limited support for self-awareness of bias and inclusive communication • Absence of tools for equitable collaboration and inclusive matchmaking
Dissemination and Exploitation of Results	• Training rarely covers how to assess and maximise social, cultural, or economic impacts • Underdeveloped content on writing for non-academic audiences, including industry • Lack of formats supporting peer exchange (e.g. peer-to-peer events) and collaborative dissemination strategies
Science Communication (towards media and policymakers)	• Gaps in inclusivity and communication of uncertainty and scientific limits • Limited training on engagement with policymakers and writing effective policy briefs • Insufficient coverage of digital tools such as SEO, analytics, and data visualisation • Weak treatment of misinformation, disinformation, and pseudoscience; underrepresentation of theory, dialogic approaches, and impact tracking tools
Management and Leadership	• Lack of content on mentor-mentee relationships and leadership development for different career stages • Missing training on networking and building support systems • Limited focus on mental health, wellbeing, and emotional resilience in research careers

*Inclusivity and diversity.* Training materials frequently overlook topics central to inclusive research practice. Across several areas, general keywords such as inclusive communication, intersectionality, and inclusive matchmaking point to a lack of in-depth content. While some gender equality trainings begin to touch on broader aspects of diversity, such as generic content focused on Gender Equality Plans (GEPs), most remain general, lacking support for self-reflection on bias, action-oriented tools, or institutional implementation strategies. There is minimal attention to cultural competence, inclusive language, and the integration of local or indigenous knowledge systems. Intersectionality—which refers to how different aspects of a person’s identity (such as gender, race, class, and disability) intersect to shape their experiences and potential disadvantages—is also underrepresented, both as a framework and in practical application, particularly in citizen science, research integrity, and science communication.

*Training approaches.* Many resources rely on static formats and transmissive pedagogies. Active learning strategies—such as problem-based and project-based learning, co-creation, and train-the-trainer formats—are inconsistently used, despite their potential to increase engagement and knowledge retention. There is limited integration of peer-to-peer events, collaborative tools, gamification elements, and interactive components. Modularity and hybrid learning designs are often absent, and restrictive licensing or lack of metadata hampers adaptation and reuse.

*Disciplinary and sectoral reach.* A strong disciplinary skew is evident in the distribution of training, with the natural sciences and technical fields dominating. There are potentially unmet training needs in the humanities, social sciences, medical sciences, and civic-sector engagement. Research integrity, data management, and citizen science training, for instance, rarely include discipline-adapted materials or examples beyond science and technology. While our mapping focused on resources for students, researchers, and research support staff, there appears to be limited openly accessible training tailored to non-academic stakeholders such as industry and the private sector—likely because such offerings are often delivered as internal or paid courses within organisations.

*Advanced-level offerings.* Resources tailored to advanced learners and senior researchers remain limited in number. Few training resources articulate skill progression beyond the introductory level, and most lack frameworks for distinguishing foundational, intermediate, and expert-level competencies. Concepts such as leadership, mentoring, and institutional change management are rarely addressed, even though they are critical for embedding Open RRI practices in research cultures.

*Policy, ethics, and impact orientation.* Several training areas do not engage much with societal and policy contexts. Dissemination and exploitation training often neglect the social, cultural, or economic impact of research, while science communication training leaves out policy engagement, communication of uncertainty, and misinformation. Open access materials seldom cover policy mandates or legal risks, and research integrity training often overlook supervision ethics, power dynamics, environmental sustainability, AI, and the social consequences of misconduct. FAIR data training lacks comprehensive guidance on metadata standards, curation, and ethical data handling, particularly for non-specialist audiences. Intellectual property rights are inconsistently addressed across training types.

*Language and accessibility.* English dominates the landscape of available training resources, creating barriers for non-native speakers and researchers in under-resourced regions, although as mentioned above this is partly a bias in the training resources mapped by the project members. Nonetheless, the lack of multilingual materials and translation support limits reach and reuse. Technically, many resources fall short on accessibility: they lack clear structures, supportive metadata, and modular design, and are often constrained by restrictive copyright licences. These factors collectively hinder the broader uptake, localisation, and long-term sustainability of Open RRI training.

## Discussion

This study responds to a well-documented need for enhanced training in transferrable skills to support Open RRI globally, while also promoting researcher employability ( CoARA, 2022; European Commission, 2023; European Commission: Directorate-General for Research and Innovation et al., 2022; Marschalek et al., 2017; Kohrs et al., 2023). We have mapped and quality assessed existing Open RRI training resources for researchers. Our objective was to enhance the visibility and reusability of these resources—especially those that are openly accessible digitally—identify existing gaps and ultimately contribute to a more dynamic and sustainable training landscape for Open RRI. The mapping and assessment revealed a landscape that is both fragmented and uneven, with key challenges relating to the scope, depth, customisation, pedagogical approaches, inclusivity, accessibility, and long-term sustainability of available training—issues that are explored in more detail in the sections below.

### Fragmentation and consolidation in the training landscape

Over 500 training resources were identified across key Open RRI skill areas, including individual training opportunities, curated catalogues, and resource collections—many originating from previous collaborative European initiatives. Despite this volume, the training landscape remains highly fragmented. Many resources are hosted on project-specific platforms that are not maintained over time, impeding discoverability and long-term usability. This fragmentation makes it difficult for researchers to locate relevant training and for institutions to embed high-quality resources into their programmes.

To address this, more sustained coordination is needed to federate resources across platforms and enable systematic access. Initiatives like OpenAIRE’s OpenPlato and the EU-Citizen.Science platform provide promising models. They not only host diverse training offers but also promote their visibility and reuse through communities of practice. Centralising access while maintaining openness and modularity could enhance the long-term impact of publicly funded training efforts.

### Gaps in depth, scope, and customisation

The quality assessment and SWOT analysis highlighted significant gaps in training coverage. Thematic gaps were especially apparent in the areas of regulatory frameworks (e.g. Plan S), societal engagement, intersectionality, and the inclusion of indigenous or local knowledge systems. Likewise, citizen science, data stewardship, and research ethics training rarely included advanced-level or action-oriented components. Most training pays minimal attention to leadership development, mentoring, or skills tailored to senior researchers, despite evidence from both the literature and our study that, senior researchers play a crucial role in mentoring and advancing Open RRI ( Tijdink et al., 2021). Training specifically targeting this group remains limited — discussions during our mutual learning events highlighted that later-career researchers are often underrepresented in training activities, with participants suggesting these opportunities could be more effectively framed as networking events or peer-led discussions facilitated by Open RRI champions.

From a policy, ethics, and impact orientation, training often lacks depth in areas such as data governance, the ethical implications of emerging technologies, and practical tools for stakeholder involvement. Disciplinary and stakeholder gaps also persist: most training continues to target early-career researchers in the natural and technical sciences, with limited attention to the social sciences, humanities, health fields, or non-academic stakeholders such as industry actors and policy professionals. In science communication, for example, the question of how to address the needs of “occasional, active, and professional science communicators” remains unresolved ( Lewenstein & Baram-Tsabari, 2024). Research Integrity training offers a rare counterexample of career-stage customisation, with initiatives such as Path2Integrity ( van den Hoven et al., 2023), VIRT ^2^UE ( Evans, Marušić et al., 2021; Evans, Schmolmueller et al., 2023), and ENERI ( Penders et al., 2018) targeting learners from PhD students to principal investigators and research ethics committee members. Yet outside of such cases, tailored approaches remain scarce, underscoring the need for more diversified training development across domains and stakeholder groups.

A key challenge relates to balancing discipline-agnostic and domain-specific content. While many research integrity issues are cross-cutting and aim to foster critical thinking across disciplines ( Pizzolato et al., 2020), case studies and examples often disproportionately focus on the biomedical sciences. A similar pattern is seen in FAIR data training, which frequently adopts a generic format, despite the need for discipline-specific guidance on standards, ontologies, file formats, and repositories ( García, Berberi, et al., 2022). Customised resources—such as GDPR training tailored to social scientists or ethics guidance developed for biomedical researchers—demonstrate the added value of content that moves beyond general recommendations. More targeted and diversified training development is needed to ensure relevance across research domains and sectors.

### Pedagogical quality and interactivity

Our study identified a variety of training formats designed to accommodate diverse learning preferences, including face-to-face, virtual, synchronous, and asynchronous delivery, as well as static materials such as webinars and guidebooks. In asynchronous courses, forums and occasional synchronous sessions can help foster interaction. Each format offers specific benefits and poses distinct challenges—with hybrid formats reported as the most difficult to implement in ways that ensure equal participation and engagement.

Despite this variety, many current training resources remain anchored in transmissive, lecture-based approaches, which limits their ability to support deeper learning or behavioural change. Insights from our mutual learning events, survey, and desk research highlighted the effectiveness of active learning strategies—a finding that aligns with Freeman et al. (2014), who demonstrated the impact of active learning on educational outcomes.

Approaches such as project-based learning, co-creation, scenario analysis, and game-based learning have proven particularly effective in areas like research integrity, science communication, and gender equality training. For instance, game-based learning has been applied successfully in research integrity education through tools such as the Dilemma Game ( Erasmus University Rotterdam, 2020), and in gender training to promote self-awareness. In science communication, experiential approaches that integrate practice and theory through iterative feedback loops have also shown strong potential ( Kankaria et al., 2024; Llorente & Revuelta, 2023).

### Inclusivity, language, and accessibility

The inclusivity of training is essential to its accessibility, effectiveness, and long-term sustainability. However, our study found a strong dominance of English-only resources, often lacking modularity or open licences for adaptation. This creates barriers to participation for non-Anglophone communities and risks reinforcing patterns of marginalisation, especially in contexts shaped by structural inequality or colonial legacies ( Zwart et al., 2024).

Translation tools offer a partial solution, but more deliberate strategies are needed to promote linguistic and epistemic diversity. Training should also engage with cultural competence, inclusive communication, and equitable collaboration—areas currently underrepresented in available materials. Expanding the reach and relevance of Open RRI training demands a stronger commitment to pluralism and a rethinking of what counts as valid knowledge.

A notable concern for inclusivity is the limited integration of intersectionality across Open RRI training resources. While some materials address diversity in terms of gender or disability, few recognise how overlapping identities—such as race, class, gender, sexuality, and disability—shape individuals’ experiences in research environments. This omission can result in training that fails to acknowledge or address the specific barriers faced by minoritized or multiply marginalised groups. Without intersectional awareness, efforts to promote inclusive research practices risk reinforcing existing inequalities or overlooking the needs of certain communities ( Zachariassen et al., 2023).

### Pathways to sustainability and integration

Sustainability emerged as a major concern in our analysis, both in technical and institutional terms. Technically, training resources must be designed according to FAIR principles ( García, Batut et al., 2020), using metadata standards ( IEEE, 2020) and adaptable formats (e.g. SCORM) to facilitate adaptation and personalisation for different audiences (Advanced Digital Learning Initiative, 2009). Licensing also matters: Open educational resources require permissive licences (e.g. CC-BY) that allow for reuse and adaptation. This is often misunderstood when repurposing materials originally created for publication rather than education.

Institutional sustainability involves embedding Open RRI training into academic curricula, professional development pathways, and institutional strategies ( Kohrs et al., 2023). This is especially important for senior researchers, whose engagement remains limited despite their influential role. Our findings echo recent calls to reframe training as networking or peer-led engagement to better appeal to this group ( Tijdink et al., 2021).

Communities like OpenAIRE and EU-Citizen.Science play a vital role in promoting both sustainability and reuse. Their platforms not only host and promote materials but also facilitate ongoing exchange between developers and users. Aligning training development with the needs of these communities—while enabling institutional uptake—can help move Open RRI training from fragmented offerings to sustainable practice.

## Recommendations

As outlined in the introduction, the ambitions of Open RRI—and their operationalisation through the ResearchComp framework—require systematic training that equips researchers with the knowledge, skills, and values to practice Open RRI across all career stages. Our mapping and assessment show that while there is growing provision, the training landscape remains fragmented, uneven, and limited in scope. Building on the six overarching themes identified in the results—scope, depth, customisation, pedagogical approaches, inclusivity, accessibility, and sustainability—we propose the following recommendations to strengthen the training ecosystem:

• *Scope and relevance:* Develop and iteratively refine a structured progression of learning outcomes that addresses the needs of learners at foundational, intermediate, and more advanced levels. Training should respond to locally identified concerns and priorities while simultaneously engaging with emerging issues and global challenges through interdisciplinary approaches. This will enable the inclusion of diverse stakeholder perspectives and promote context-sensitive learning.
• *Depth and progression:* Articulate clear pathways for progression from foundational to advanced levels, including leadership development, mentoring, and skills for later-career researchers. Generic, beginner-level courses provide a starting point, but deeper engagement requires domain-specific tools, advanced case studies, and action-oriented training.
• *Customization:* Balance discipline-agnostic content with domain-specific examples. While cross-cutting principles can be taught broadly, effective application requires training tailored to the vocabularies, file formats, standards, and ethical dilemmas of disciplines.
• *Pedagogical approaches:* Move beyond transmissive, lecture-based models towards active, interactive formats such as project-based learning, co-creation, scenario analysis, and gamification. Training formats should cater for diverse learning preferences (face-to-face, online, hybrid, synchronous, asynchronous) while ensuring equal opportunities for participation and engagement.
• *Inclusivity and accessibility:* Promote inclusivity by integrating intersectional perspectives and engaging with underrepresented groups. Training should be offered in multiple languages to reduce overreliance on English and should recognise and incorporate diverse epistemologies, including indigenous and local knowledge systems. Accessibility should also extend to cost—ensuring that resources are openly available rather than paywalled or restricted to internal institutional use.
• *Sustainability:* Foster long-term sustainability through both technical and social mechanisms. Technical strategies include making training materials FAIR, adopting recognised metadata and file standards, and applying permissive licensing to support adaptation and reuse. Social mechanisms include embedding training in academic curricula, engaging with communities of practice, and supporting train-the-trainer initiatives to build local expertise and extend impact across institutions and regions.

## Ethics and consent

This study received ethical approval the Research Ethics Committee (Institutional Review Board) at Aarhus University (Approval no. 2023–001). The study was carried out in compliance with Aarhus University guidelines, Horizon Europe ethical principles, and applicable data protection regulations, including the General Data Protection Regulation (GDPR). All participants were informed about the study and participated voluntarily. Informed consent was obtained prior to participation: electronically for the survey and verbally (following prior written information) for interviews. Data were handled confidentially and in accordance with GDPR and institutional data protection policies.

## Use of AI language assistance

During the preparation of this work, the authors used ChatGPT-5 to assist with language-related tasks such as spelling, grammar, synonym suggestions, and sentence reformulation. All content generated or modified using the tool was subsequently reviewed and edited by the authors. The authors take full responsibility for the final content of the publication.

## Data Availability

The data underlying this study consist of the mapped training resources and associated metadata collected and analysed within the PATTERN project. The full dataset is openly available in Zenodo as a spreadsheet: https://doi.org/10.5281/zenodo.17198972 ( Lagido et al., 2025). The content theme vocabulary used in the mapping process and to enable analysis of coverage, gaps, and opportunities is available as extended data: https://doi.org/10.5281/zenodo.20396499 ( PATTERN Consortium, 2026a). Additional structured summaries of the mapped training areas, which complement the dataset and provide detailed descriptive analysis, are available as extended data: https://doi.org/10.5281/zenodo.20340672 ( PATTERN Consortium, 2026b). The interview guide and informed consent materials used for data collection are also available as extended data: https://doi.org/10.5281/zenodo.20341278 ( Lagido & Nielsen, 2026). Data are available under the terms of the Creative Commons Attribution 4.0 International.
