# Supplementary material for: Training resources on open and responsible research and innovation (Open RRI): gaps, quality and opportunities to advance training
Source: Open Res Eur. 2026 Jun 23;6:217. [Version 1] doi: 10.12688/openreseurope.23510.1 (PMC13560565; doi:10.12688/openreseurope.23510.1)
Supplement: Peer review history

# Reviewer report: Reviewer response for version 1

**DOI:** 10.21956/openreseurope.25451.r76671

**Published:** 2026-08-10

**Authors:** Angela Simone, Anna Pellizzone

**Affiliations:** 1 Fondazione Giannino Bassetti ETS, Milan, Italy

The article presents findings from the PATTERN project, which mapped and assessed the quality of Open RRI training resources across Europe. Drawing on desk research, a survey, 12 interviews, 5 data workshops and 3 mutual learning events, the authors collected 571 training resources across 8 skill areas and assessed the quality of a subset of them using a quality framework based on EU-Citizen.Science, a H2020-SwafS project. The training landscape they describe is fragmented, largely at an introductory level, and mainly in English. The paper closes with six recommendations covering scope, depth, customisation, pedagogy, inclusivity and sustainability

 This is solid work. The scale of the mapping and the mix of methods give it real value, and the Zenodo dataset holds up well, offering reusable metadata for all 571 resources under an open licence.

 A few points below would be worth addressing to strengthen the paper further.

 The resources chosen for the quality assessment were selected because they already looked well structured, accessible and reusable, as the methods section states openly. This is a reasonable choice given the constraints of the project, but it means the assessed resources are not representative of the whole landscape, closer to a best-case sample. The results then describe training resources as performing well across most criteria without noting that this applies only to the assessed subset, and this finding is later used to describe Open RRI training more broadly. We would ask the authors to state clearly, wherever these results are discussed, that they describe the assessed subset rather than the full pool of 571 resources.

 A few procedural details are missing that a reader would need in order to judge the findings: how many people were invited to complete the survey and what the response rate was, which matters given that the survey was distributed through the project's own network and that two thirds of respondents came from public research institutes; and how the quality ratings were carried out, including how many people rated each resource and whether any check of agreement between raters was performed.

 There is also a literature gap worth noting. The introduction relies on the European Commission's five dimension model of RRI but does not cite Owen, von Schomberg and Macnaghten (2021), who reflect on a decade of RRI and argue that it became a set of disconnected components, unevenly applied across Europe, a picture close to what this paper itself finds. The same paper also discusses directly how Open Science relates to RRI, which is precisely the connection this paper's own concept of Open RRI is built on, so it would be worth citing on that point as well. It would also help to briefly discuss where the term Open RRI comes from, since it does not appear to exist outside EU funded projects.

 On the disciplinary balance raised in the discussion, we would not treat the focus on the natural and technical sciences as a problem in itself, since RRI developed mainly within that space. The gap worth flagging is narrower: a shortage of discipline specific examples in research integrity, data management and citizen science training for fields such as the humanities and social sciences, rather than the overall balance across disciplines.

 It is not entirely clear how the authors define citizen science for the purposes of this study, and specifically whether it is meant to also cover broader forms of public or citizen engagement. This matters because the European Commission's own related work, including the 2022 Mutual Learning Exercise on citizen science initiatives and the 2024 Mutual Learning Exercise on public engagement in research and innovation, treats citizen science as one specific form of engagement within the wider public engagement landscape, rather than as an overarching category. A short clarification from the authors on how the term is used and bounded in this mapping would help.

 The introduction also lists specific SwafS projects as evidence that the field is active, without explaining why these projects were chosen over others. RRI Tools, an earlier and well known initiative, is not mentioned, nor is work on territorial RRI or on RRI in the private sector, and it is unclear whether a specific time frame guided the selection.

 The recommendations sit somewhat apart from the rest of the paper and would probably read better folded into the discussion, as direct implications of the findings, rather than kept as a separate list of actions. As written, they are grounded in the findings but remain fairly general: the recommendation on inclusivity and accessibility calls for rethinking what counts as valid knowledge without specifying how this might be done, and the first recommendation bundles two distinct ideas, learning progression and local relevance, that would work better split into two points or merged with the following recommendation on depth and progression.

 Overall, this is a useful and well-conducted mapping study that adds real value to the Open RRI training field. In our view, the paper can be recommended for approval, and addressing the points raised above would further strengthen it.

If applicable, is the statistical analysis and its interpretation appropriate?

Yes

Is the study design appropriate and is the work technically sound?

Yes

Is the work clearly and accurately presented and does it engage with the current literature?

Yes

Are the conclusions drawn adequately supported by the results?

Partly

Are sufficient details of methods and analysis provided to allow replication by others?

Partly

Are all the source data and materials underlying the results available?

Yes

Reviewer Expertise:

Responsible Research and Innovation, Citizen Engagement in Research and Innovation, Open Science, Science Communication

We confirm that we have read this submission and believe that we have an appropriate level of expertise to confirm that it is of an acceptable scientific standard.

# Reviewer report: Reviewer response for version 1

**DOI:** 10.21956/openreseurope.25451.r76673

**Published:** 2026-07-22

**Authors:** Ralf Lindner

**Affiliations:** 1 The Fraunhofer Institute for Systems and Innovation Research, Karlsruhe, Germany

Lagido et al. present findings from the Horizon Europe project PATTERN, examining the landscape of training resources on Open and Responsible Research and Innovation (Open RRI) across Europe. Employing a mixed-methods approach, combining desk research, surveys, semi-structured interviews, data workshops, and mutual learning events, the authors mapped and quality-assessed 571 training resources across eight transferable skill areas: Open Access, FAIR Data, Citizen Science, Research Integrity, Gender/Non-discrimination/Inclusion, Dissemination and Exploitation, Science Communication, and Management and Leadership. Quality assessment was adapted from the EU-Citizen.Science framework, applying a five-point rating scale complemented by SWOT analysis. Results indicate a fragmented and uneven training landscape dominated by introductory-level, English-language resources, with limited provision at intermediate and advanced levels. Recurring weaknesses include poor adaptability, restrictive licensing, lack of multilingual accessibility, and insufficient coverage of topics such as intersectionality, policy engagement, supervision ethics, and indigenous knowledge. The authors conclude that coordinated, inclusive, and institutionally embedded approaches are needed to advance sustainable Open RRI capacity building.

 The article makes a valuable and timely contribution to the field of Open RRI by providing a comprehensive mapping of training resources. The scope of the study (571 resources across eight skill areas, involving 19 institutions from 13 countries) is impressive. The mixed-methods design is a particular strength: by combining desk research, surveys, semi-structured interviews, data workshops, and mutual learning events, the authors achieve a meaningful triangulation of evidence that enhances the credibility and depth of their findings. The adaptation of the EU-Citizen.Science quality framework, supplemented by a SWOT analysis, provides a transparent and structured basis for evaluating training resources. The identification of thematic gaps is especially useful. The paper is well-written and logically structured.

 Despite these strengths, the paper also shows a few areas for improvement:

 The introduction states that the eight skill areas were selected "based on their centrality to Open RRI," yet the rationale for this selection remains largely asserted rather than argued. Given that the entire study is structured around these eight domains, a more explicit and literature-grounded justification is warranted. For instance, why are these eight areas prioritized over other potentially relevant competencies, such as open methodology, reproducibility, or public engagement beyond citizen science? Without such justification, readers may question whether the mapping is comprehensive or subject to selection bias.

 The methodology section describes the various data collection instruments in reasonable detail but falls short in explaining how training resources were systematically identified and selected, particularly in the desk research phase. Key questions remain unanswered: Which databases, platforms, or repositories were searched? Were predefined search terms or Boolean strings used? How was inter-rater reliability ensured across the distributed team of project partners? What steps were taken to mitigate the acknowledged language bias towards English-language resources? Without this information, it is difficult to assess the completeness and replicability of the mapping exercise. The authors are encouraged to provide a more explicit account of the search and selection procedure to allow readers and future researchers to critically evaluate and build upon the methodology.

 The recommendations section is arguably the weakest part of an otherwise strong paper. While the six recommendations are logically structured and clearly grounded in the empirical analysis, they remain at a high level of abstraction and largely reiterate findings already discussed in the preceding sections. Given the richness of the data collected, readers would benefit considerably from more operationalized guidance. For example, the recommendation on *sustainability* could specify concrete institutional mechanisms (e.g., integration into doctoral training agreements or national research strategy frameworks), while the recommendation on *inclusivity and accessibility* could elaborate on practical approaches to multilingual resource development, such as community translation models or co-design with non-Anglophone partners. Expanding each recommendation with targeted, actionable steps would substantially increase the paper's practical utility for training providers, research institutions, and policymakers.

 All in all, this is a well-executed and relevant study that addresses an important gap in the Open RRI literature. From my point of view, the paper can be recommended for indexing subject to minor to moderate revisions.

If applicable, is the statistical analysis and its interpretation appropriate?

Yes

Is the study design appropriate and is the work technically sound?

Yes

Is the work clearly and accurately presented and does it engage with the current literature?

Yes

Are the conclusions drawn adequately supported by the results?

Yes

Are sufficient details of methods and analysis provided to allow replication by others?

Partly

Are all the source data and materials underlying the results available?

Yes

Reviewer Expertise:

Expertise in the analysis of science and innovation policy and governance, governance of research performing organisations, RRI.

I confirm that I have read this submission and believe that I have an appropriate level of expertise to confirm that it is of an acceptable scientific standard.
